# A Single Fast Test for Semicircular Canal Dehiscence—oVEMP n10 to 4000 Hz—Depends on Stimulus Rise Time

**DOI:** 10.3390/audiolres12050046

**Published:** 2022-08-24

**Authors:** Ian S. Curthoys, Ann M. Burgess, Leonardo Manzari, Christopher J. Pastras

**Affiliations:** 1 Vestibular Research Laboratory, School of Psychology, the University of Sydney, Sydney, NSW 2006, Australia; 2 MSA ENT Academy Center, 03043 Cassino, Italy; 3 Faculty of Engineering and Science, Macquarie University, Sydney, NSW 2109, Australia

**Keywords:** otolith, vestibular, oVEMP, utricular, clinical audio vestibular testing, vestibular screening test

## Abstract

As previously reported, a single test measuring oVEMP n10 to 4000 Hz stimuli (bone-conducted vibration (BCV) or air-conducted sound (ACS)) provides a definitive diagnosis of semicircular canal dehiscence (SCD) in 22 CT-verified patients, with a sensitivity of 1.0 and specificity of 1.0. This single short screening test has great advantages of speed, minimizing testing time, and the exposure of patients to stimulation. However, a few studies of the 4000 Hz test for SCD have reported sensitivity and specificity values which are slightly less than reported previously. We hypothesized that the rise time of the stimulus is important for detecting the oVEMP n10 to 4000 Hz, similarly to what we had shown for 500 and 750 Hz BCV. We measured oVEMP n10 in 15 patients with CT-verified SCD in response to 4000 Hz ACS or BCV stimuli with rise times of 0, 1, and 2 ms. As a result, increasing the rise time of the stimulus reduced the oVEMP n10 amplitude. This outcome is expected from the physiological evidence of guinea pig primary vestibular afferents, which are activated by sound or vibration. Therefore, for clinical VEMP testing, short rise times are optimal (preferably 0 ms).

## 1. Introduction

A defect, such as thinning or absence of the bony casing of the semicircular canal, is referred to as a semicircular canal dehiscence (SCD) ([1,2] see Wackym et al. [3] for a recent extensive review). SCD results in changes in the mechanical operation of the labyrinth [4,5], which causes characteristic symptoms (such as dizziness, autophony, etc.). However, the symptoms are idiosyncratic, and thus, an objective indicator of the SCD is required for diagnosis. Clinically, this has meant the use of vestibular evoked myogenic potentials (VEMPs) to sound or vibration. VEMPs are small myogenic potentials recorded in response to ACS or BCV (see [6] for a review). The ocular VEMP (oVEMP) is predominantly due to the activation of utricular type I receptors and irregular primary afferents from the striola of the utricular macula [7,8,9].

Experiments on guinea pigs recording single primary vestibular afferent neurons, which are activated by sound or vibration stimulation, have shown that these are calyx-bearing irregular primary afferents from the striola of the otolithic maculae [8,9]. Moreover, these receptors and afferents have very precise and short latencies to transient BCV and ACS, such as brief tone bursts or clicks. Therefore, they are extremely sensitive to the very onset of the stimulus envelope [10]. Increasing the rise time of the stimulus reduces that onset transient, and thus, reduces the effectiveness of the stimulus for activating the receptors and irregular afferents which are responsible for VEMPs [8]. In audiology, the term, rise time, refers to the time from the onset of the stimulus to the maximum amplitude of the stimulus. Therefore, considering these neural results, a short rise time should be ideal for activating the type I receptors and irregular primary afferents, and thus, causing an oVEMP n10.

The oVEMP is particularly valuable in identifying patients with a dehiscence of a semicircular canal. Compared to VEMPs in healthy subjects, the amplitude of the oVEMP increases and the threshold for establishing the oVEMP decreases in SCD patients [11,12]. By comparing the oVEMP n10 amplitude in patients with CT-verified SCD to the n10 amplitude in healthy subjects, Manzari et al. showed that a useful cut-off criterion for diagnosing SCD was an oVEMP n10 to 500 Hz BCV greater than 10 µV (baseline to peak) [12]. Therefore, at the Cassino clinic, 95% of healthy subjects have an oVEMP n10 amplitude lower than 10 µV when stimulated by 500 Hz BCV stimulus with the clinical standard of 1 ms rise time. This criterion, with 10 µV oVEMP n10, had a sensitivity of 1.0 and specificity of 0.97 for detecting SCD in the group of CT-verified patients. As a result, it was recommended that patients with an oVEMP n10 amplitude greater than 10 µV should be suspected of possibly having an SCD.

The problem with this single criterion of n10 greater than 10 µV to 500 Hz Fz BCV is that some perfectly healthy subjects show an oVEMP n10 which is quite large, but the subjects are completely asymptomatic [12]. The ideal diagnostic indicator would be a stimulus, in which healthy subjects have an extremely poor or absent response. In the interim, it has been reported that many phenomena apart from semicircular canal dehiscence can cause enhanced oVEMP n10 to 500 Hz stimulation (e.g., even intracochlear schwannoma [13]), and thus, the amplitude of the response to 500 Hz is not specific for SCD, which is why the use of 4000 Hz to identify SCD was developed (variants of the 4000 Hz test are now referred to as “high frequency VEMPs“ [14]). A method with few stimulus presentations is preferred, and in 2013, we reported that a single test consisting of only 50 presentations of 4000 Hz stimuli, BCV or ACS, had excellent diagnostic accuracy. In 2013, Manzari et al. showed that 22 patients with a CT-verified SCD had clear oVEMP to 4000 Hz, whereas 27 healthy subjects did not show a detectable oVEMP n10 to this very high frequency [15]. Therefore, a single test averaging the response to only 50 stimuli at 4000 Hz is a very valuable initial screening test for patients with suspected SCD [15,16]. Recent developments have been refined to optimize both sensitivity and specificity of high frequency cVEMP and oVEMP for detecting SCD and it has been argued that “VEMP tests using high frequency stimuli are the most accurate single diagnostic test for SCD” [17,18].

Other authors have confirmed the high specificity and sensitivity of high frequency stimuli to be a highly effective way of identifying SCD [14,17,19]. However, there have been some reports where the measured specificity and sensitivity of the 4000 Hz test for SCD has been lower than what was found by Manzari et al. [19,20].

There are several possible reasons for this reduced result, and one reason is that some studies with lower sensitivity and specificity have used long rise times. As we will explain below, the new neural evidence shows that the response of primary vestibular afferent neurons to sound and vibration stimuli which are responsible for VEMPs, is activated by the rate of change of the BCV or ACS envelope. The increasing rise time reduces the rate of change, and thus, would be expected to reduce the amplitude of the oVEMP n10 response to 4000 Hz. Therefore, this reduces the sensitivity and specificity of the 4000 Hz test. In a study by Lin et al., the authors used a very long rise time of 4 ms [20]. To test this hypothesis, we examined 15 patients with CT-verified SCD and measured their oVEMPs to both 4000 Hz ACS and BCV with rise times of 0, 1 or 2 ms, with the prediction that increasing the rise time should cause smaller oVEMP n10 potentials. In some patients, we also tested the same rise times with 8000 Hz, which had oVEMP n10 potentials. This has shown to be almost as effective for SCD detection as 4000 Hz in the earlier study [15].

## 2. Materials and Methods

All of the subjects gave informed consent for inclusion prior to study participation, which was conducted in accordance with the Declaration of Helsinki. The protocol was approved by the MSA ENT Academy Center Institutional Review Ethics Committee 03-2014.

In total, there were 15 patients including 7 males and 8 females, ranging in age from 27 to 81 with an average age of 55 years. The participants were tested with informed consent (see Table 1 for patient demographics). The inclusion criterion was that these patients needed to have been tested previously and demonstrated enhanced oVEMP n10, and thus had received CT scans, which confirmed their SCD. Only patients with CT-verified unilateral SCD were included.

**Figure 1:**
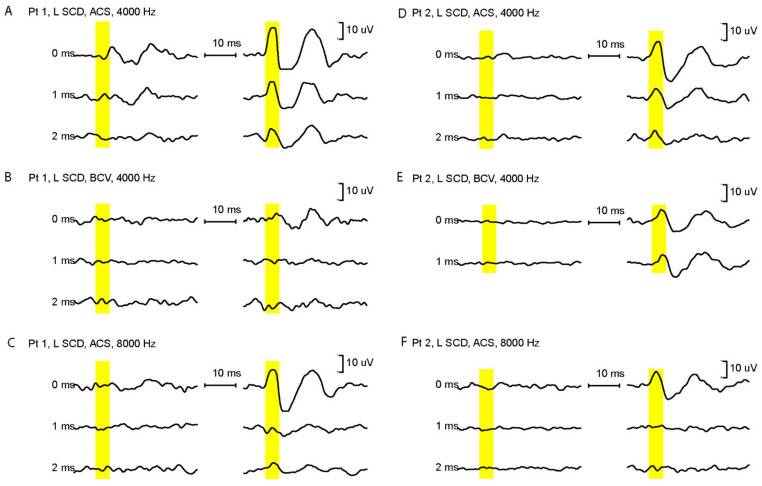
(**A**–**F**) Examples of the oVEMP n10 in response to 4000 Hz BCV and ACS stimuli at rise times of 0, 1, and 2 ms for two patients. The yellow bars define the regions for the oVEMP n10. The oVEMP for the SCD ear is shown in the right columns. Increasing the rise time from 0 to 2 ms decreases the amplitude of the oVEMP n10. In these patients, we also tested the same rise times with 8000 Hz ACS stimulus (**C**,**F**). While a rise time of 0 ms at 8000 Hz generates a clear oVEMP in both patients, prolonging the rise time to only 1 ms abolishes the oVEMP n10. These results confirm that events at the very onset of the high frequency stimulus are of major importance in initiating the oVEMP n10.

We measured the averaged oVEMP n10 component, in response to 50 presentations of short tone bursts (at 4/s) of binaural ACS delivered by Telephonics TDH49 headphones at 120 dB SPL or BCV delivered by a hand-held Bruel and Kjaer mini-shaker 4810 to the midline of the forehead at the hairline (a location called Fz), at a force level of around 130 dB FL re 1 µN (see Figure 1) [21]. Surface EMG electrodes beneath both eyes recorded the oVEMP n10 for both eyes simultaneously. The stimulus frequency was 4000 Hz and the rise times, plateau times, and fall times were 0-2-0, 1-2-1, and 2-2-2 ms with a zero-crossing start. Moreover, we tested some patients at 8000 Hz with the same rise, plateau, and fall times. The patients did not find these frequencies and intensities uncomfortable.

For recording oVEMPs, the patient was laid supine on a bed with their head supported on a pillow but positioned with the chin pitched slightly nose down. During each test, they were required to look straight up (toward the top of their head) to a fixation point at approximately 25° above their visual straight ahead and during stimulation to maintain fixation on that fixation dot, located approximately 60 to 70 cm from their eyes. The standard oVEMP montage was used: For each eye, the active (+) electrode was positioned on the infraorbital ridge 1 cm below the lower eyelid, and the reference (−) electrode about 2 cm below the active electrode [21]. The electrodes were aligned with the pupil as the subject looked directly upward. Unrectified EMG was amplified and sampled at 20 kHz, band-pass was filtered between 3 and 500 Hz, and 50 trials were averaged with an ICS Chartr EP 200 device (Otometrics, Denmark). Means and two-tailed 95% confidence intervals were calculated, and the level of statistical significance was set at *p* < 0.05 [22].

## 3. Results

**Figure 2:**
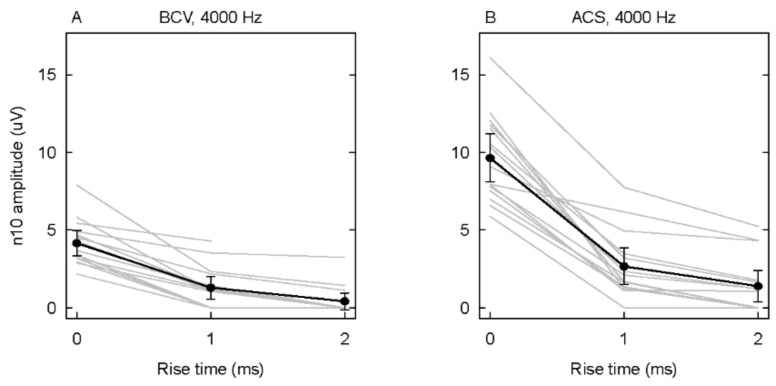
The averaged oVEMP n10 to 4000 Hz ((**A**) for BCV and (**B**) for ACS) at each rise time for individual patients (gray lines) and the mean across patients in black lines with error bars as 95% confidence intervals.

In SCD patients, increasing the rise time decreases the amplitude of oVEMP n10 to both ACS and Fz BCV stimulation. An example of the raw data for two CT-verified SCD patients in response to 4000 Hz tone bursts with varying rise times is shown in Figure 1. It is clear that the amplitude of the n10 component of the oVEMP decreases, for both ACS and BCV 4000 Hz stimuli, as the rise time increases (Figure 1 and Figure 2). These decreases are statistically significant. The additional data using 8000 Hz show how the n10 is dependent on the rise time.

## 4. Discussion

The results demonstrate that the rise time is a particularly crucial factor in determining the amplitude of oVEMP n10 to 4000 Hz stimulation in patients with CT-verified SCD. Specifically, the stimuli with short rise times are more effective in generating oVEMP n10 than stimuli with long rise times. Moreover, the very earliest part of the stimulus is critical for generating n10. This result follows from recent clinical and neural data published in [15], in which the otolithic receptors and afferents responsive to sound and vibration are sensitive to the very onset of the stimulus envelope, as we will explain below.

### 4.1. Clinical Neurophysiology-Healthy Subjects

To answer the following question, “Is there other evidence that it is the early part of the stimulus which is critical for generating VEMPs?”, Lim et al. showed the major importance of the first few milliseconds of a 500 Hz BCV stimulus. This was performed by systematically reducing the duration of the stimulus and measuring the amplitude of oVEMP n10 response at decreasing stimulus durations (with 0 ms rise time) in healthy subjects [23]. The reduction in duration was from 10 to 2 ms and the onset acceleration was identical for all durations. The surprising result was that this reduction in duration (which reduces the energy delivered immensely), caused no reduction in oVEMP n10 amplitude. In fact, the n10 amplitude increased at 2 ms duration: The average amplitude at 10 ms was 0.9 µV ± 1.6 (SD) and the amplitude at 2 ms was 1.4 µV ± 1.9 [23]. In unpublished observations, we have confirmed this surprising result and extended it to show that even a stimulus of only 1 ms duration elicits an oVEMP n10 as large as the one produced by a 10 ms stimulus. This is very surprising since subjectively the stimuli are completely different—a 10 ms 500 Hz vibration is a strong vibration, whereas a 1 ms stimulus is very weak and feels like a light touch. However, this very short duration stimulus with an abrupt rise time, is as effective as a long duration stimulus in generating VEMPs. This result is very strong evidence that the very earliest part of the stimulus is crucial for eliciting oVEMPs.

**Figure 3:**
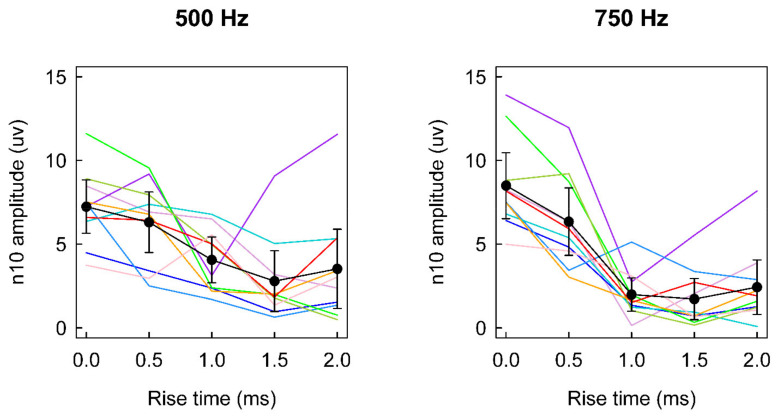
The oVEMP n10 amplitude as a function of stimulus rise time for 10 healthy subjects tested with tone bursts of BCV delivered to Fz with the 4810 mini-shaker at frequencies of 500 and 750 Hz. Traces show the data for individual subjects. The black trace in each panel shows the mean over 10 subjects. Error bars show 95% confidence intervals of the mean. Data replotted from [27].

Complementing that result is other evidence from studies in healthy subjects measuring the amplitude of n10 as the rise time of 500 Hz or 750 Hz short tone bursts was varied. The data for BCV are shown in Figure 3, which indicates that as the rise time was increased from 0 to 2 ms, there was a very large reduction in the amplitude of n10 of about 50% for 500 Hz and even more for 750 Hz. Similarly, Kantner et al. reported that in healthy subjects, for a 500 Hz ACS stimulus a rise time of 4 ms produced around 30% smaller oVEMPs on average than the 0 ms rise time [24].

In healthy subjects, and also for ears with normally encased labyrinths (the healthy ear in SCD patients), 4000 Hz does not elicit an oVEMP n10 [12] (see the records for the healthy ear in Figure 1). This is probably due to the fact that primary semicircular canal afferent neurons are not activated by 500 Hz or 4000 Hz ACS or BCV stimuli (as demonstrated in recordings of vestibular afferents in guinea pigs with normally encased labyrinths) [25]. However, after an SCD, these previously unresponsive irregular semicircular canal neurons show strong, phase-locked activation by both ACS and BCV stimuli [10], which extends to high frequencies. Therefore, these physiological studies show that one reason for the existence of a clear oVEMP n10 to 4000 Hz is that after SCD this stimulus not only enhances the response of irregular otolithic afferents [25], but also activates irregular semicircular canal afferents, which are unresponsive to high frequencies when the bony labyrinth is intact [10,25,26]. In response to Fz BCV after SCD, the stimulus activates both otolithic and canal afferents, both of which phase lock to very high frequencies [10]. Therefore, the oVEMP n10 in SCD patients is a result of activation of both otolithic and canal irregular afferents.

The frequencies of 4000 and 8000 Hz are significantly higher than the usually considered frequencies that stimulate otolithic receptors and afferents. However, there is excellent evidence that these high frequencies do activate otolithic receptors and afferents. In guinea pigs, the measure of receptor response (the utricular microphonic (analogous to the cochlear microphonic)) shows that utricular receptors do respond to these high frequencies after cochlear ablation. Therefore, there is no contribution to the utricular microphonic response from cochlear receptors. The microphonic response shows clear responses up to above 3 kHz [28]. Recordings from single primary afferent neurons with irregular resting discharge show that after an SCD, primary otolithic and semicircular canal neurons can be activated by sound and vibration at stimulus levels used for clinical testing [10,25,26]. The most compelling evidence is that opening the bony casing of the canal causes previously unresponsive semicircular canal neurons to be activated by ACS and BCV. Whereas they are not activated at levels used for clinical testing when the canal is encased in bone. This is strong evidence that the oVEMP n10 is driven by afferent input from both otolithic and semicircular canals.

### 4.2. The Importance of Stimulus Jerk for VEMPs

Jones et al. have studied the VsEP in several species. This is the neural potential measured from the scalp, which is triggered by abrupt pulses of linear acceleration, and thus, largely otolithic [29]. The authors have shown this otolithic compound action potential s dependent on the rate of change of the linear acceleration stimulus rather than the magnitude of the linear acceleration itself. This is evidence that otolithic receptors and irregular afferents are responsible for generating VEMPs, which are activated by the change in linear acceleration at stimulus onset rather than only linear acceleration.

Finally, very recent data have recorded the vestibular compound action potential (a measure of the synchronous activation of many primary afferent neurons), which is recorded near the vestibular nerve in guinea pigs in response to brief BCV and ACS transients. Recording directly above the utricular macula in guinea pigs in response to a transient BCV stimulus shows that increasing the rise time of the transient stimulus has a very substantial effect on the synchronous activation of receptors and afferents, reducing the amplitude of the vestibular compound action potential as the rise time is increased [30]. The long rise time acts to “smear” the synchronous activation of otolithic afferents, and thus, greatly reduces the amplitude of the vestibular compound action potential to BCV clicks.

### 4.3. Rise Time in Clinical VEMP Testing

When air-conducted tone bursts were originally demonstrated to elicit VEMPs over tensed sternocleidomastoid muscles, standard audiometric parameters were used for the investigation—1 or 2 ms rise time for the 500 Hz test stimulus [31]. These rise times are appropriate for audiometry, where it is essential to have a slow rise time to eliminate an audible click at stimulus onset. However, they are precisely the opposite of what is required for eliciting responses from vestibular receptors and afferents since a slow rise time reduces the rate of change of the stimulus, which is the key parameter of the stimulus that activates the receptor hair cells and afferents, and thus, causes VEMPs.

Studies of the 4000 Hz test in SCD patients have used a variety of rise times, including some very long rise times, even 4 ms as used in the Lin et al. study [20]. Tran et al. [19] and Batuecas-Caletrio et al. [14] have both used 2 ms and our data indicate that with these rise times it is likely that there would be no detectable oVEMP n10 in some patients. We consider that the lower sensitivity and specificity values for the 4000 Hz test that the authors reported, may have been due in part to the fact that they did not use an optimum rise time for the 4000 Hz stimuli.

The international recommendations for VEMP testing report a number of parameters which are optimal for eliciting VEMPs, but unfortunately the rise time is not given sufficient attention [11,32]. As it is clear from these results, the high frequency VEMP test should use the shortest rise time possible. Unfortunately, different audiometers use different means of setting the rise time of the stimulus (some allow the operator to set the rise time to an exact value). Whereas others define the rise time according to the stimulus frequency, for example, since many cycles of the stimulus frequency are from zero to maximum. Notably, however the rise time is set; thus, it is important to maintain the rise time to the absolute minimum that the audiometer or stimulus generator allows. In our opinion, the duration of the rise time should be reported with the VEMP results. Unfortunately, some audiometers cannot generate stimuli with 0 ms rise time. However, it is important for a clinician to realize that the magnitude of the oVEMP measured with a stimulus with a long rise time underestimates the true amplitude of the oVEMP n10.

Future research should examine whether other labyrinthine conditions, which generate enhanced oVEMPs (such as intracochlear schwannomas) also cause enhanced oVEMP n10 to 4000 Hz stimulation.

## 5. Conclusions

In this study, the evidence demonstrates that, once again, 4000 Hz is an excellent test for identifying SCD. We fully agree with the statement by Noij et al. that “VEMP tests using high frequency stimuli are the most accurate single diagnostic test for SCD“ [17,18].

Increasing the rise time of stimulus to 4000 Hz in VEMP testing of patients with CT-verified SCD decreases the size of the n10 component of the oVEMP. To optimize the 4000 Hz screening test for SCD, it is suggested that the rise time should be kept as short as possible and preferably at 0 ms.

Patients with CT-verified SCD are scarce, thus the number of patients in this study was small, with a total of only 15. However, in this study, as in our previous study [15], every single CT-verified SCD patient showed an oVEMP n10 to the 4000 Hz stimuli with short rise times (15 patients in this study, 26 patients in our previous study, for a total of 41 patients with 100% detection). This is convincing evidence that 4000 Hz with a short rise time is an excellent screening test for SCD [18]. As we have shown, the result is in close accordance with the evidence from the physiology of the effect of SCD on canal afferent neuron responses. Given the variability of the thickness of the bony casing of semicircular canals between different individuals, it is possible that the occasional asymptomatic healthy individual may show a response to 4000 Hz. Moreover, the clinician should be aware of this possibility, which, in our view does not detract from the recognized value of 4000 Hz with a short rise time as a simple fast screening test for SCD [18].

Given that sound and vibration activate both canal and otolithic neurons, the explanation for the enhanced response in SCD patients is that it is due to the activation of both utricular and semicircular canal afferents after SCD. The canal contribution is supported by evidence from human patients after SCD: In SCD patients, the axis of the eye movement generated by the ACS stimulus corresponds to the anatomical axis of the dehiscent canal [33].

## Figures and Tables

**Table 1:** Patient demographics.

Patient	Gender	Age	Affected Side
1	F	75	left
2	M	75	right
3	M	62	left
4	M	81	left
5	M	51	right
6	M	39	left
7	F	46	right
8	M	32	right
9	F	27	left
10	F	51	right
11	F	39	left
12	F	57	right
13	M	75	left
14	F	59	left
15	F	61	left

## Data Availability

The raw data supporting the conclusions of this paper will be made available by the authors, without undue reservation.
